# A novel diagnostic model for predicting immune microenvironment subclass based on costimulatory molecules in lung squamous carcinoma

**DOI:** 10.3389/fgene.2022.1078790

**Published:** 2022-12-14

**Authors:** Fangfang Duan, Weisen Wang, Wenyu Zhai, Junye Wang, Zerui Zhao, Lie Zheng, Bingyu Rao, Yuheng Zhou, Hao Long, Yaobin Lin

**Affiliations:** ^1^ Department of Medical Oncology, State Key Laboratory of Oncology in Southern China, Collaborative Innovation Center for Cancer Medicine, Sun Yat-Sen University Cancer Center, Guangzhou, China; ^2^ Department of Thoracic Surgery, The First Affiliated Hospital of Shantou University Medical College, Shantou, China; ^3^ Department of Thoracic Surgery, State Key Laboratory of Oncology in Southern China, Collaborative Innovation Center for Cancer Medicine, Sun Yat-Sen University Cancer Center, Guangzhou, China; ^4^ Lung Cancer Research Center, Sun Yat-Sen University, Guangzhou, China; ^5^ Medical Imaging Division, Department of Medical Imaging and Interventional Radiology, State Key Laboratory of Oncology in Southern China, Collaborative Innovation Center for Cancer Medicine, Sun Yat-Sen University Cancer Center, Guangzhou, China

**Keywords:** costimulatory molecules, lung squamous carcinoma, machine learning algorithm, tumor immune microenvironment, diagnostic biomarker

## Abstract

There is still no ideal predictive biomarker for immunotherapy response among patients with non-small cell lung cancer. Costimulatory molecules play a role in anti-tumor immune response. Hence, they can be a potential biomarker for immunotherapy response. The current study comprehensively investigated the expression of costimulatory molecules in lung squamous carcinoma (LUSC) and identified diagnostic biomarkers for immunotherapy response. The costimulatory molecule gene expression profiles of 627 patients were obtained from the The Cancer Genome Atlas, GSE73403, and GSE37745 datasets. Patients were divided into different clusters using the k-means clustering method and were further classified into two discrepant tumor microenvironment (TIME) subclasses (hot and cold tumors) according to the immune score of the ESTIMATE algorithm. A high proportion of activated immune cells, including activated memory CD4 T cells, CD8 T cells, and M1 macrophages. Five CMGs (FAS, TNFRSF14, TNFRSF17, TNFRSF1B, and TNFSF13B) were considered as diagnostic markers using the Least Absolute Shrinkage and Selection Operator and the Support Vector Machine-Recursive Feature Elimination machine learning algorithms. Based on the five CMGs, a diagnostic nomogram for predicting individual tumor immune microenvironment subclasses in the TCGA dataset was developed, and its predictive performance was validated using GSE73403 and GSE37745 datasets. The predictive accuracy of the diagnostic nomogram was satisfactory in all three datasets. Therefore, it can be used to identify patients who may benefit more from immunotherapy.

## Introduction

Lung cancer is the second most commonly diagnosed tumor and is the most frequent cause of cancer-related mortality worldwide (Sung et al., 2021). Lung squamous carcinoma (LUSC) is one of the major subtypes of lung cancer, which accounts for approximately 25%–30% of all lung cancer cases (Singh et al., 2021). Due to hardly any driver gene observed in most patients with LUSC, LUSC patients can obtain limited benefits from targeted therapy (Miller and Hanna, 2021).

In the last few years, immune checkpoint inhibitors (ICIs) including the programmed cell death protein 1 (PD1), programmed cell death ligand 1 (PD-L1), and the cytotoxic T-lymphocyte antigen 4 (CTLA4) inhibitors, have rapidly changed the therapeutic landscape of different malignancies, such as melanoma, lung carcinoma, and nasopharyngeal cancer (Isaacs et al., 2021). As well as the NSCLC, several large randomized controlled trials, such as KeyNote-407, KeyNote-042, CheckMate-017, and OAK studies support ICIs as first-line therapy for advanced LUSC (Brahmer et al., 2015; Rittmeyer et al., 2017; Paz-Ares et al., 2018; Mok et al., 2019). Besides, CheckMate-816 trial also proved immunotherapy can prolong progression-free survival for local advanced NSCLC (Forde et al., 2021).

Despite the unprecedented durable response rates achieved with ICIs, most patients with LUSC do not benefit from cancer immunotherapy (*de novo* resistance) and will even develop recurrence after the initial response (acquired resistance) (Insa et al., 2021; Shang et al., 2021). Identifying indicator to select candidates benefitting from immunotherapy has become a hot area. PD-L1 is the most commonly used indicator for identifying candidates for ICI treatment in clinical practice. Different clinical trials assessed its predictive value and associations between its expression and major pathological response. KeyNote-042 study enrolled patients with PD-L1 expression ≥1% and found only patients with PD-L1 expression ≥50% obtained survival benefit from pembrolizumab (Mok et al., 2019). CheckMate-816 trial revealed that patients with PD-L1 expression ≥50% had higher complete pathological rate than other patients in immunotherapy arm (Forde et al., 2021). However, the use of PD-L1 as a biomarker had some limitations. A multicenter observational study demonstrated PD-L1 expression is not an indicator for LUSC patients with first-line pembrolizumab (Doroshow et al., 2021). In addition, spatial and temporal heterogeneity and inconsistent binary cutoff point of PD-L1 expression also limit its use as a biomarker (Mino-Kenudson et al., 2022). Tumor mutational burden (TMB), which is another potential predictor of response to combined immunotherapy, has been explored. Based on the KeyNote-189, pembrolizumab was approved for treatment of patients with advanced solid tumors (including LUSC) with TMB>10, while CheckMate-026 also found the association between TMB and survival (Carbone et al., 2017; Garassino et al., 2020). Nevertheless, the predictive value of TMB was only showed in ICI monotherapy and lost in immunochemotherapy (Sholl et al., 2020). Besides TMB may be inconsistent when using different targeted gene panels or whole exome sequencing (Mino-Kenudson et al., 2022). And the issue of inconsistent binary cutoff point is also a limitation of TMB (Garassino et al., 2020). Therefore, novel predictors of treatment response should be identified to appropriately select patients who can receive immunotherapies with the aim to design individualized strategies.

The era of immunotherapy has promoted the increasing parse of immune system function and immunosuppressive status generated in the tumor immune microenvironment (TIME), which comprised multiple immune, stromal, and mesenchymal cells, cytokines, and chemokines (Singh et al., 2020; Zhang et al., 2020). TIME plays an important role in the processes of tumor initiation, progress, development, and metastasis (Binnewies et al., 2018). Naïve T cells are activated *via* two indispensable signals before they attack neoantigens, among which is the non-specific costimulatory signal (Bluestone, 1995). Tumor cells commonly release wrong messages to T cells and block the recognition of costimulatory signals by altering costimulatory molecular structures and expressions in TIME. Further, they induce an immunosuppressive TIME (Sanmamed and Chen, 2019), which is characterized by exhaustion and anergy of T cells. Subsequently, immunosuppressive TIME could enable tumor cells to evade host immune-mediated elimination (Hanahan and Weinberg, 2011). Costimulatory molecules include the B7-CD28 family. Among them, eight members (CD80, CD86, PD-L1, PD-L2, ICOSLG, B7-H3, B7x, and HHLA2) are classified under the B7 family, and five molecules (CD28, CTLA4, ICOS, PD-1, and TMIGD2) belong to the CD28 family (Janakiram et al., 2015). The tumor necrosis factor (TNF) family has 19 molecules belonging to the TNF ligand superfamily (TNFSF) and 29 molecules to the TNF receptor superfamily (TNFRSF) (Croft and Siegel, 2017). Costimulatory molecules play an important role in immune cell proliferation, differentiation, activation, survival, and functions (Kraehenbuehl et al., 2021). Moreover, they can be possible novel targets or can be added to the current immunotherapeutic regiments (Croft et al., 2013; Schildberg et al., 2016). The predictive model based on costimulatory molecule genes (CMGs) for the individual predictions of prognosis and immunotherapy response has been explored in multiple tumors, including lung adenocarcinoma, prostate cancer, and head and neck squamous cell carcinoma (Zhang et al., 2020; Aye et al., 2021; Ge et al., 2021). However, their functions and predictive value in LUSC remain poorly understood. Thus, there is a need to systematically explore CMGs in patients with LUSC.

Therefore, to explore the predictive value of the abovementioned 60 CMGs, the current study aimed to analyze LUSC gene expression profiles using datasets collected from The Cancer Genome Atlas (TCGA) and the Gene Expression Omnibus (GEO) databases (Zhang et al., 2020). Via k-means clustering, patients with LUSC were divided into two different immune subclasses (cold and hot tumors). Next, the CIBERSORT algorithm (Newman et al., 2015) was used to compare differences in 22 immune cells infiltrating the TIME between cold and hot LUSC tumors. Then, we combined two main machine learning algorithms (Least Absolute Shrinkage and Selection Operator (LASSO) (Li and Sillanpää, 2012) and Support Vector Machine-Recursive Feature Elimination (SVM-RFE) (Sanz et al., 2018), with several bioinformatic methods to screen out diagnostic biomarkers among 60 candidate CMGs in patients with LUSC. Based on these CMGs, a diagnostic nomogram for predicting individual immune environment subclasses in patients with LUSC was established, and its predictive performance and clinical value were validated.

## Materials and methods

### Extraction and standardization of gene expression dataset

We downloaded the count data and clinical information of LUSC project from the TCGA database (https://tcga-data.nci.nih.gov/tcga/), and the count data were log2 transformed for subsequent analysis. We also obtained the log2 transformed data of gene expression array from two GEO databases (https://www.ncbi.nlm.nih.gov/geo/) (GSE73403, GSE37745 and GSE93157) using the “GEO query” package. The gene expression datasets were normalized with the “SVA” and “limma” packages to remove the batch effect from biotechnology. Samples loss to follow-up or missing clinical information were excluded. The TCGA, GSE73403, and GSE37745 datasets included 492, 69, and 66 cancer samples, respectively. The GSE93157 dataset included 13 LUSC treated with immunotherapy. Five of 13 patient were progressive disease, which was defined as not response. Three patients with partial response, and five patients with stable disease were seen as response. TCGA and GSE73403 datasets were used to identify the diagnostic CMGs. The diagnostic nomogram was developed based on TCGA dataset, GSE73403 and GSE37745 datasets were used for validating the nomogram. GSE93157 dataset was used for exploring the predictive value of diagnostic CMGs in immunotherapy. Further, we obtained 60 costimulatory molecules, including 13 molecules belonging to the B7-CD28 family and 47 to the TNF family, according to a previous study (Supplementary Table S1) (Zhang et al., 2020).

### Patient clustering based on CMGs

Patients with LUSC were clustered using the unsupervised consensus clustering method, the k-means machine learning algorithm with the “Cluster” R package. First, the corresponding optimal cluster numbers in all three datasets were determined using the “factoextra” R package. Moreover, we performed principal component analysis after k-means clustering using the “factoextra” R package to present the visualization results of patient clustering and to evaluate clustering efficacy. Next, the “ESTIMATE” R package (Yoshihara et al., 2013) was used to calculate and compare differences in tumor purity, immune and stromal scores among different clusters in the TCGA, GSE73403, and GSE37745 datasets. Further, patients with LUSC in these three datasets were stratified into the “hot” and “cold” tumor subclasses based on their immune and stromal scores, which could reflect tumor purity and infiltration levels of immune cells (Yoshihara et al., 2013).

### Landscape of immune cells infiltrating the TIME

We analyzed the standardized CMG expression profiles of the TCGA, GSE73403, and GSE37745 datasets and used the CIBERSORT algorithm with perm set to 1000 to explore the landscape of 22 immune cells infiltrating (Newman et al., 2015) the TIME between patients in the “hot” and “cold” tumor subclasses. Besides, another algorithm, microenvironment cell populations-counter (MCP-counter) was also performed to estimate abundance of 10 types of cells between “hot” and “cold” tumor subclasses.

### Functional annotation and pathway enrichment analyses

Gene set enrichment analysis (GSEA, https://www.gsea-msigdb.org/gsea/index.jsp) was conducted on patients with LUSC in the “hot” *versus* “cold” tumor subclasses *via* the Java GSEA (version 4.0.1) using the Kyoto Encyclopedia of Genes and Genomes (KEGG) pathway in C2 and Gene Ontology (GO) terms in C5 to perform the functional annotation and potential enrichment analyses (Subramanian et al., 2005). The false discovery rate was set at < 0.25, and a *p*-value of <0.05 was normalized as significant enrichment.

### Identification of diagnostic biomarkers among candidate CMGs

In the TCGA and GSE73403 datasets, we initially utilized the LASSO logistic regression method with the “glmnet” R package to screen out diagnostic biomarkers among candidate CMGs at the optimal value of log lambda with the smallest classification error (35). In addition, the SVM-RFE machine learning algorithm based on the support vector machine was used to identify the best diagnostic biomarkers from all candidate CMGs by subtracting the feature vector determined using SVM with the “e1071” and “caret” packages. Then, we overlapped diagnostic biomarkers identified using the abovementioned two machine learning algorithms with the “scMerge” package, and the same CMGs in the abovementioned two datasets were finally utilized in the logistic regression analysis to identify the CMG biomarkers.

### Development and validation of the diagnostic nomogram based on CMG biomarkers

Using the abovementioned CMG biomarkers, a diagnostic nomogram was developed in the TCGA training dataset using the “rms” R package for predicting individualized TIME subclasses among patients with LUSC. The predictive performance and clinical value of this CMG-based diagnostic nomogram was further assessed and validated using the receiver operating characteristic (ROC) and calibration curves both in the TCGA training and validation datasets.

### Statistical analysis

The CMG expression profiles of patients with LUSC in these three datasets were listed as raw and standardized. The TCGA dataset was defined as the training cohort and the GSE73403 and GSE37745 datasets as the validation cohort. SVM model using sigmoid kernel function was performed to investigate the diagnostic value of CMGs in anti-PD1 therapy. All machine learning algorithms and bioinformatic analyses were performed using the R software (version 4.0.1, Vanderbilt University, Nashville, TN). A *p*-value of <0.05 was considered statistically significant unless specified otherwise.

## Results

### Gene expression dataset acquisition and standardization

**FIGURE 1:**
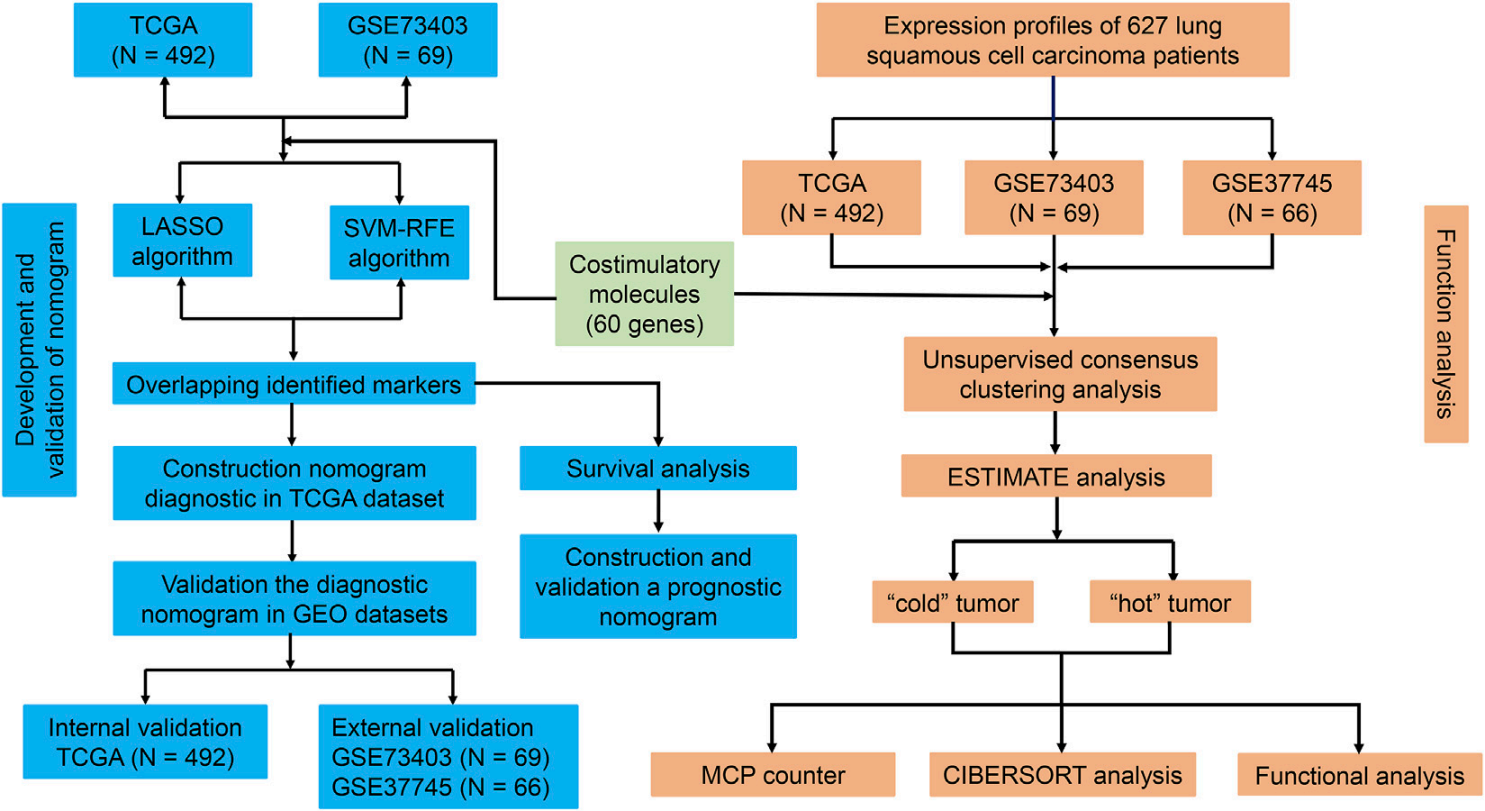
Flowchart of the study design.

Figure 1 shows the flow chart of the current study. We annotated the gene expression profiles of the three datasets. Then, all 60 CMGs were merged with these microarray matrixes. Next, the TCGA dataset included 492 LUSC samples (59 CMGs), and the GSE73403 and GSE37745 datasets comprised 69 (57 CMGs) and 66 (58 CMGs) LUSC samples, respectively. Then, the “SVA” and “limma” R packages were used to standardize the expression profiles of CMGs in the three datasets. Finally, there were still 55 CMGs available for the subsequent analysis, which included 12 members belonging to the B7-CD28 family and 43 members to the TNF family.

### Patient clustering based on CMGs

**FIGURE 2:**
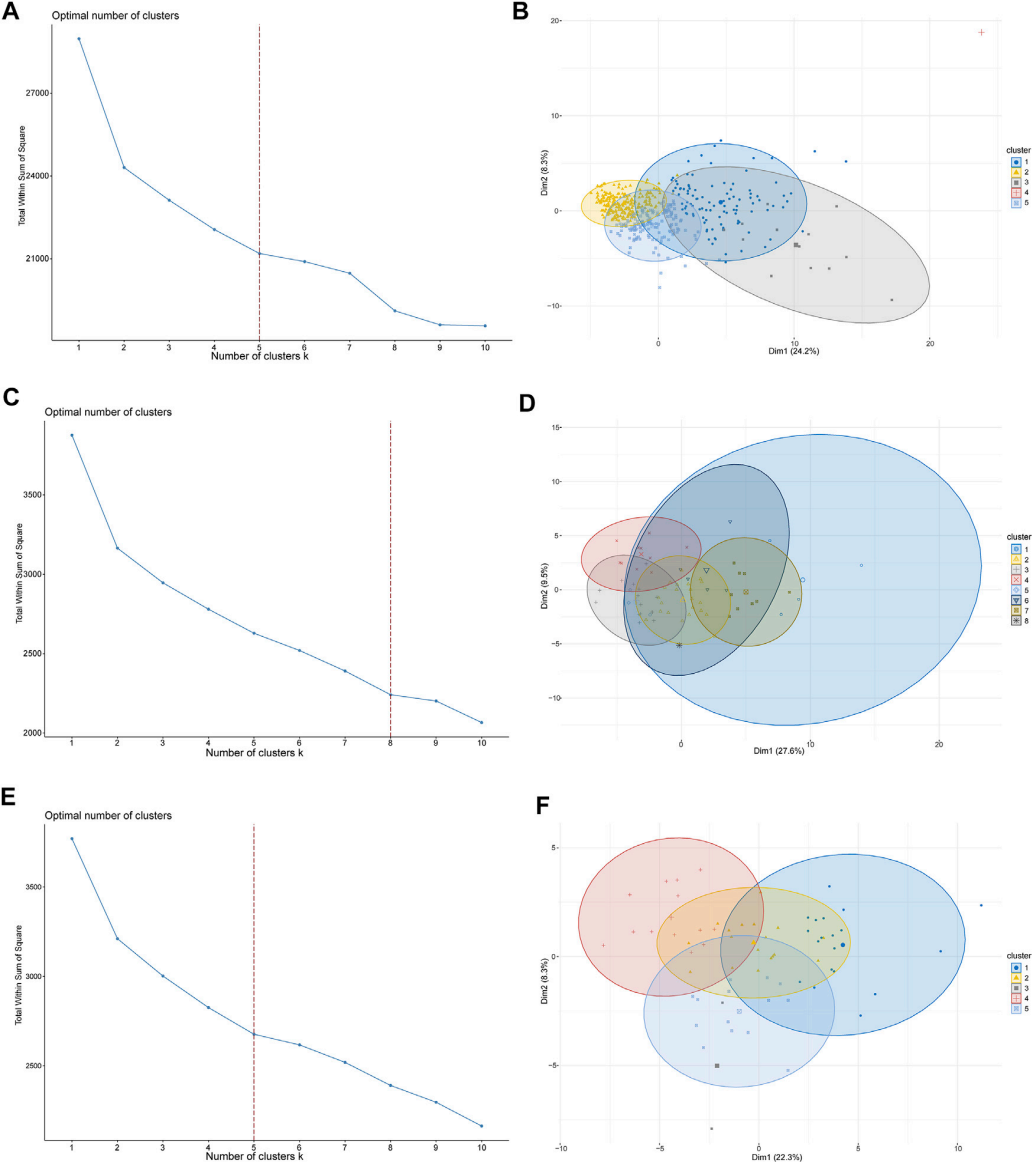
LUSC patient-clustering based on costimulatory molecule genes (CMGs). **(A)** The curve of the total within the sum of squared error curve for the corresponding cluster number *k* in TCGA dataset; **(B)** The principal component analysis (PCA) plot of clustered patients in TCGA dataset; **(C)** The curve of the total within the sum of squared error curve for the corresponding cluster number *k* in the GSE73403 dataset; **(D)** The PCA plot of clustered patients in the GSE73403 dataset; **(E)** The curve of the total within the sum of squared error curve for the corresponding cluster number *k* in the GSE37745 dataset; **(F)** The PCA plot of clustered patients in the GSE37745 dataset.

To investigate the potential functions and clinical value of the CMGs in patients with LUSC, the unsupervised consensus clustering methods were used for patient classification. The optimal cluster numbers of the three datasets were determined *via* k-means clustering analysis, which was visualized as curves of the total of the sum of squared error for the corresponding cluster numbers of *k*. Figures 2A, C, E depict that *k* values of 5, 8, and five were the best for the TCGA, GSE73403, and GSE 37745 datasets, respectively. Next, principal component analysis was performed to assess the credibility of these cluster numbers. Principal component analysis showed that patients with LUSC could be classified into five different clusters at *k* = 5 in the TCGA dataset (Figure 2B), eight clusters at *k* = 7 in the GSE73403 dataset (Figure 2D), and five clusters at *k* = 5 in the GSE37745 dataset (Figure 2F).

**FIGURE 3:**
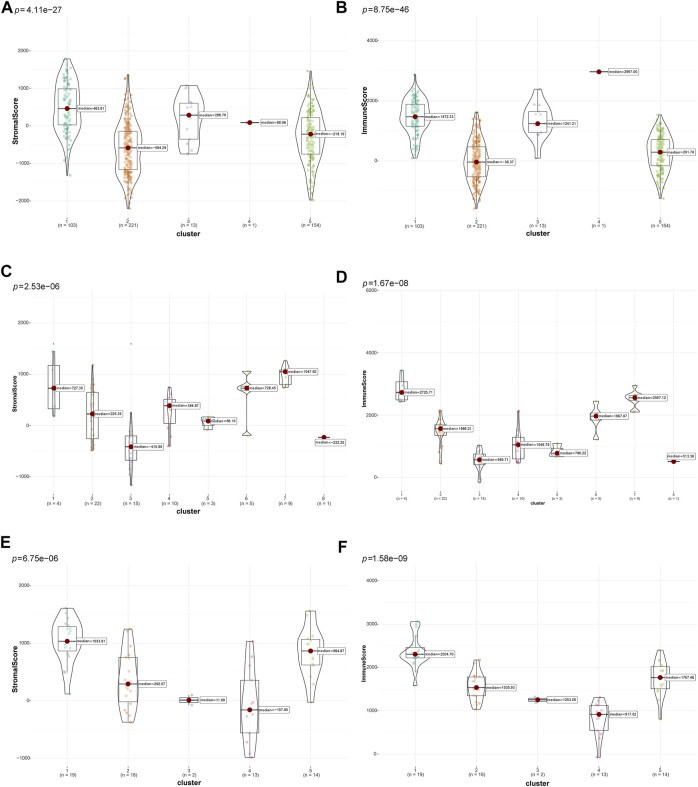
Comparison of tumor-stromal and immune scores among different LUSC patient clusters. The comparison of stromal scores **(A)** and immune scores **(B)** among different clusters in TCGA dataset; The comparison of stromal scores **(C)** and immune scores **(D)** among different clusters in the GSE73403 dataset; The comparison of stromal scores **(E)** and immune scores **(F)** among different clusters in the GSE37745 dataset.

Then, the “ESTIMATE” package (Yoshihara et al., 2013) was used to calculate and compare differences in tumor purity and stromal and immune cell infiltrations in the TIME among different clusters of patients with LUSC. The different clusters in the three datasets significantly differed in terms of tumor purity (TCGA: Supplementary Figure S1A, GSE73403: Supplementary Figure S1B, and GSE37745: Supplementary Figure S1C). In addition, there were significant differences in terms of tumor stromal and immune scores in multiple clusters in the TCGA (Figures 3A, B), GSE73403 (Figures 3C, D), and GSE37745 (Figures 3E, F) datasets. Accordingly, patients with LUSC in clusters two and five in the TCGA dataset were classified under the “cold” tumor subclass and those in clusters 1, 3, and four under the “hot” tumor subclass. In the GSE73403 dataset, patients in clusters 1,6 and seven were categorized under the “hot” tumor subclass and those in other clusters under the “cold” tumor subclass. In the GSE37745 dataset, patients in clusters two to four were classified under the “cold” tumor and those in clusters one and five under the “hot” tumor subclass.

**FIGURE 4:**
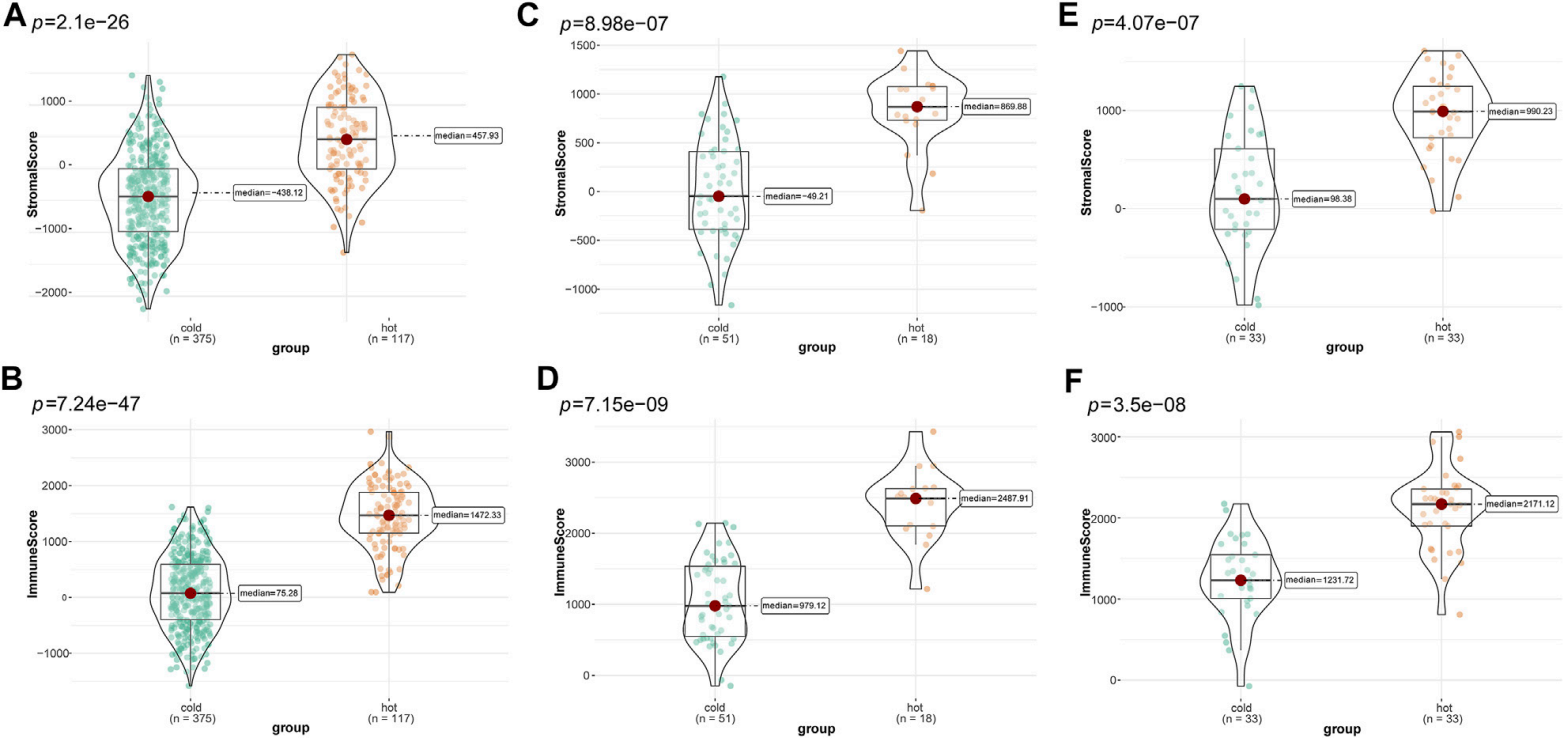
Calculation and comparison of tumor-stromal and immune scores between different TIME subclasses in LUSC, where yellow represents patients in the “hot” tumor group and green shows the patients in the “cold” tumor group. The comparison of stromal scores **(A)** and immune scores **(B)** between “hot” (clusters 1, 3, and 4) and the “cold” (clusters 2, and 5) tumor groups in TCGA dataset; the comparison of stromal scores **(C)** and immune scores **(D)** between the “hot” (clusters 1, 6, and 7)and the “cold” (clusters 2, 3, 4, five and 8) tumor groups in the GSE73403 dataset; The comparison of stromal scores **(E)** and immune scores **(F)** between the “hot” (clusters 1, and 5) and the “cold” (clusters 2 to 4) tumor groups in the GSE37745 dataset.

Subsequently, we compared differences in tumor purity and stromal and immune cell infiltrations between the “cold” and the “hot” LUSC subclasses. As shown in Figure 4, the “hot” LUSC tumors had significantly higher stromal and immune scores than the in “cold” LUSC tumors in the TCGA (Figures 4A, B), GSE73403 (Figures 4C, D), and GSE37745 (Figures 4E, F) datasets. Conversely, the tumor purity in the “cold” LUSC tumor subclasses was significantly higher than that in the “hot” LUSC tumor subclasses in the three datasets (TCGA: Supplementary Figure S2A, GSE73403: Supplementary Figure S2B, and GSE37745 Supplementary Figure S2C).

### Landscape of immune cells infiltrating the TIME

**FIGURE 5:**
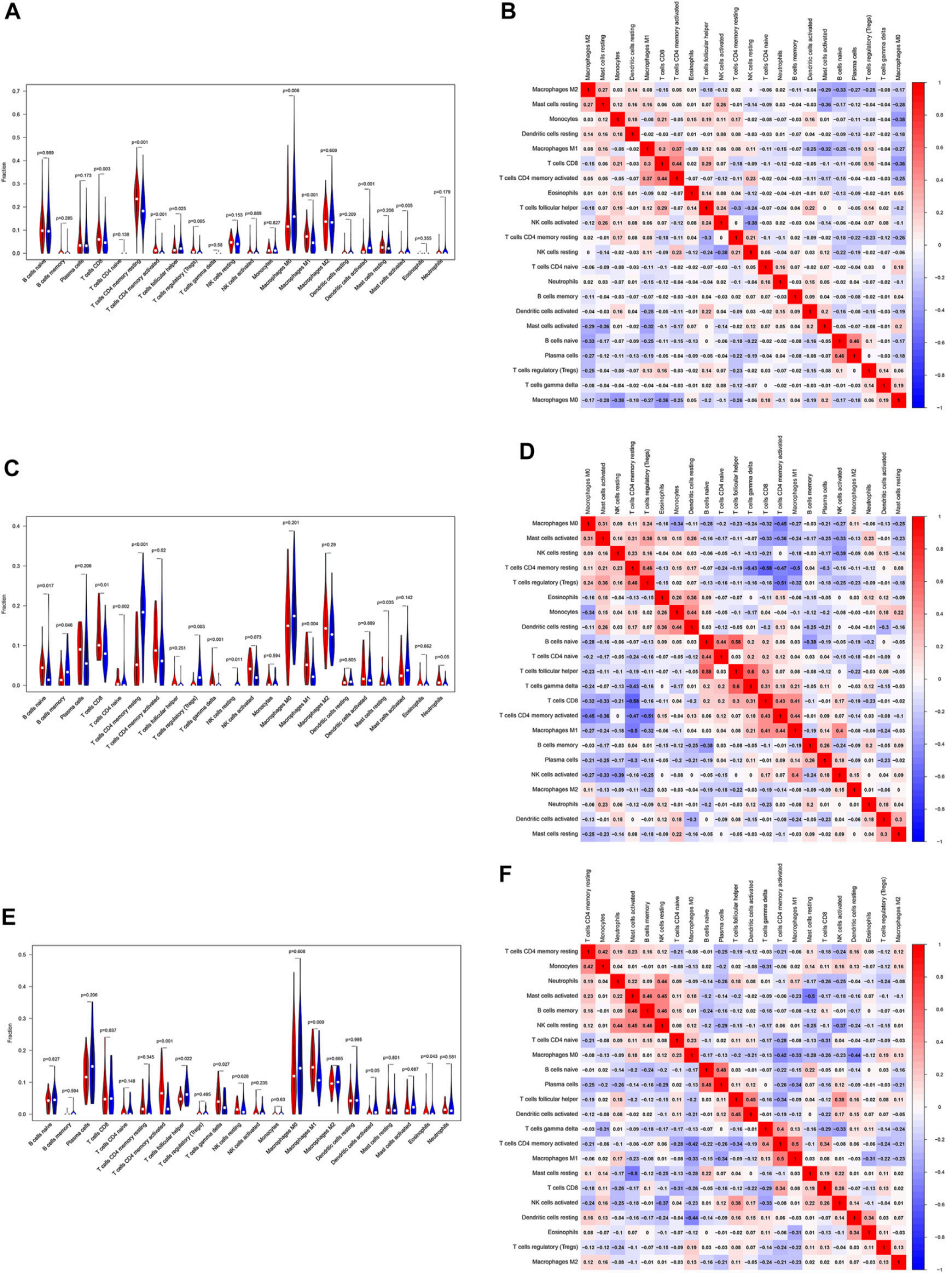
Evaluation and visualization of the 22 immune cell type infiltration landscape between different tumor groups. The left violin plot depicts infiltration disparities among immune cell types between the “hot” tumor group (red) and the “cold” tumor group (blue) in TCGA **(A)**, GSE73403 **(C)**, and GSE37745 datasets **(E)**. The correlation heat map (right) shows the correlation of immune cells between two groups in TCGA **(B)**, GSE73403 **(D)**, and GSE37745 datasets **(F)**. The number within colored squares represents the strength of the correlation; the larger is the number, the stronger is the correlation. Blue represents a negative correlation and red represents a positive correlation.

Based on the gene expression profiles, the CIBERSORT algorithm was utilized to estimate the infiltration landscape of 22 immune cells in the TIME of patients with LUSC. In the TCGA dataset, there were significant differences in the proportions of immune cells, including CD8 T cells (*p* = 0.003), CD4 memory resting T cells (*p* < 0.001), CD4 memory activated T cells (*p* < 0.001), regulatory T cells (Tregs) (*p* = 0.005), M0 macrophages (*p* = 0.006), and M1 macrophages (*p* < 0.001), between the “cold” and “hot” tumors. The percentages of CD8 T cells, CD4 memory resting T cells, CD4 memory activated T cells, Tregs, and M1 macrophages (*p* < 0.05) were higher in the “hot” than in the “cold” LUSC tumors. Meanwhile, more M0 macrophages infiltrated the “cold” tumors (*p* = 0.006) (Figure 5A). In the GSE73403 dataset, there were more significant infiltrations of naïve B cells (*p* = 0.017), CD8 T cells (*p* = 0.01), CD4 memory activated T cells (*p* = 0.02), and M1 macrophages (*p* = 0.004) in the “hot” than in the “cold” LUSC tumors (Figure 5C). In the GSE37745 dataset, the percentages of multiple immune cell infiltration, such as the CD4 memory activated T cells (*p* < 0.001), M1 macrophages (*p* = 0.009), and follicular helper T cells (*p* = 0.022), significantly differed between the “cold” and “hot” LUSC tumors. The infiltration of CD4 memory activated T cells and M1 macrophages was higher in the “hot” than in the “cold” tumors (*p* < 0.05) (Figure 5E).

In addition, we analyzed the association of 22 immune cells in LUSC tissues. The results were depicted as a correlation heatmap, as shown in Figures 5B, D, F. The number within colored squares (blue: negative, red: positive) represents the correlational strength. That is, when the number is larger, the correlation was stronger. Figure 5B shows a strong positive correlation between CD4 memory-activated T cells and M1 macrophages (Cor = 0.37), CD4 memory-activated T cells, and CD8 T cells (Cor = 0.44) in the TCGA dataset. Similar to the GSE73403 dataset, the infiltration of CD4 memory-activated T cells was positively correlated with CD8 T cells (Cor = 0.43) and M1 macrophages (Cor = 0.44) (Figure 5D). In the GSE37745 dataset, CD8 T cells (Cor = 0.50) and M1 macrophages (Cor = 0.34) were positively associated with CD4 memory activated T cells (Figure 5F).

To confirm these findings, MCP-counter was also used to investigate the 10 types of immune cells infiltrated in the TIME between “cold” and “hot” tumors. As shown in Supplementary Figure S3, almost all immune cells infiltrated more in “hot” LUSCs among three datasets, especially the anti-tumor immune cells, such asCD8 T cells (*p* < 0.001), cytotoxic lymphocytes (*p* < 0.001) and B cells (*p* < 0.001).

### Functional annotation and pathway enrichment analyses

**FIGURE 6:**
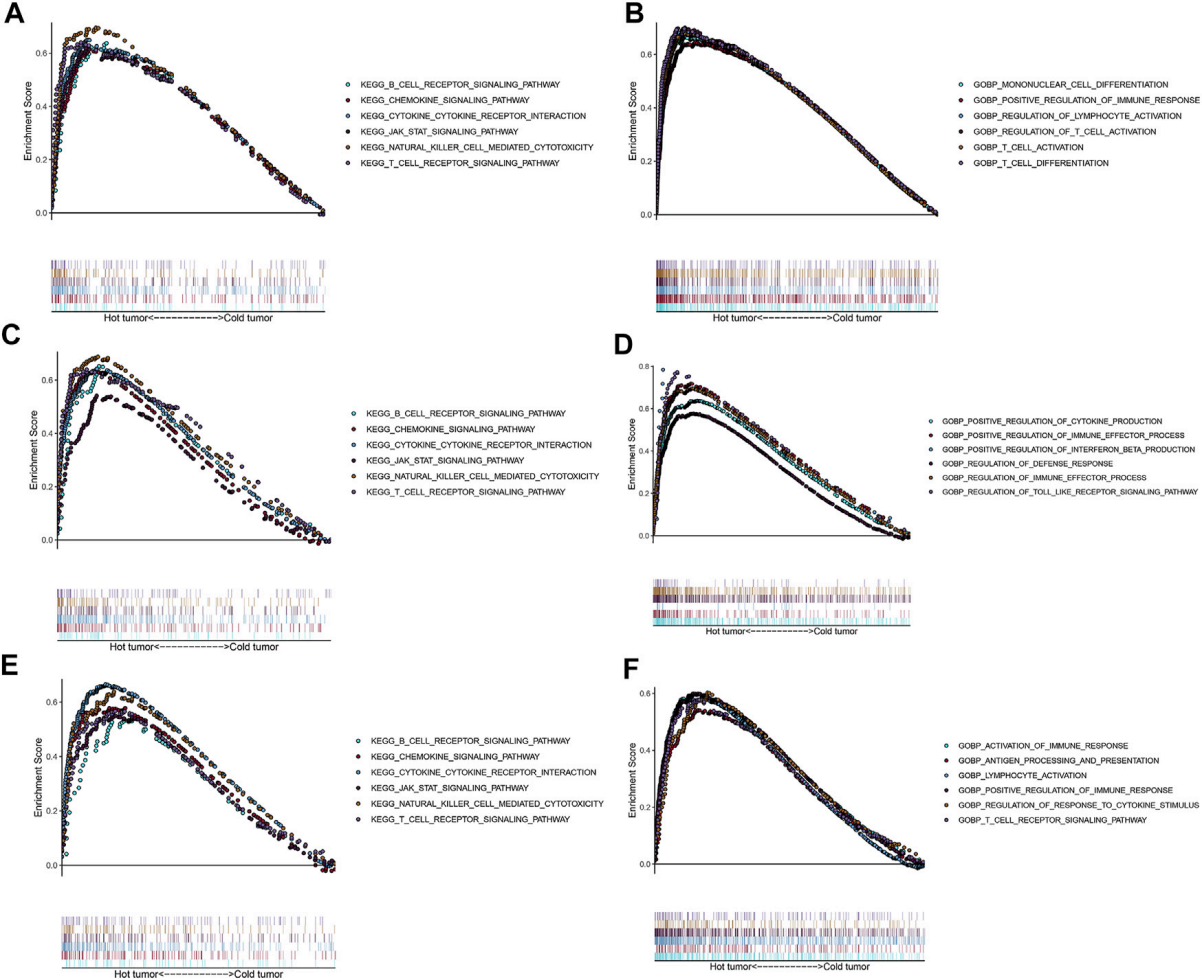
Functional analysis for the “hot” tumor and the “cold” tumor based on costimulatory molecule genes (CMGs). **(A**,**C**,**E)** The Kyoto Encyclopedia of Genes and Genomes (KEGG) enrichment analysis in TCGA, GSE73403, and GSE37745 datasets, respectively; **(B**,**D**,**F)** Gene Ontology (GO) analysis for biological processes in TCGA, GSE73403, and GSE37745 datasets, respectively.

To explore potential functions and enriched pathways among patients with LUSC, we conducted GO and KEGG enrichment analyses of the “cold” *versus* “hot” LUSC tumor subclasses. Compared with the “cold” LUSC tumors, the “hot” LUSC tumors were significantly enriched in the B and T cell receptor signaling pathways, chemokine signaling pathway, cytokine–cytokine receptor signaling pathway, JAK–STAT signaling pathway, and natural killer cell-mediated signaling pathway in the three datasets (TCGA: Figure 6A, GSE73403: Figure 6C, and GSE37745: Figure 6E). The results of GO analysis of the biological processes revealed that the “hot” LUSC tumor was mainly correlated with the regulation of T cell differentiation and activation, NK cell activation, and immune responses in the TCGA dataset (Figure 6B). In the GSE73403 dataset, the positive regulation of cytokine production and immune effector process were primarily enriched in patients with LUSC with “hot” TIME (Figure 6D). In the GSE37745 dataset, the “hot” LUSC tumors were mainly correlated with the activation of lymphocyte cells and immune response, T cell receptor signaling pathway, antigen processing and presentation, and positive regulation of immune response (Figure 6F).

### Identification of diagnostic biomarkers among candidate CMGs

**FIGURE 7:**
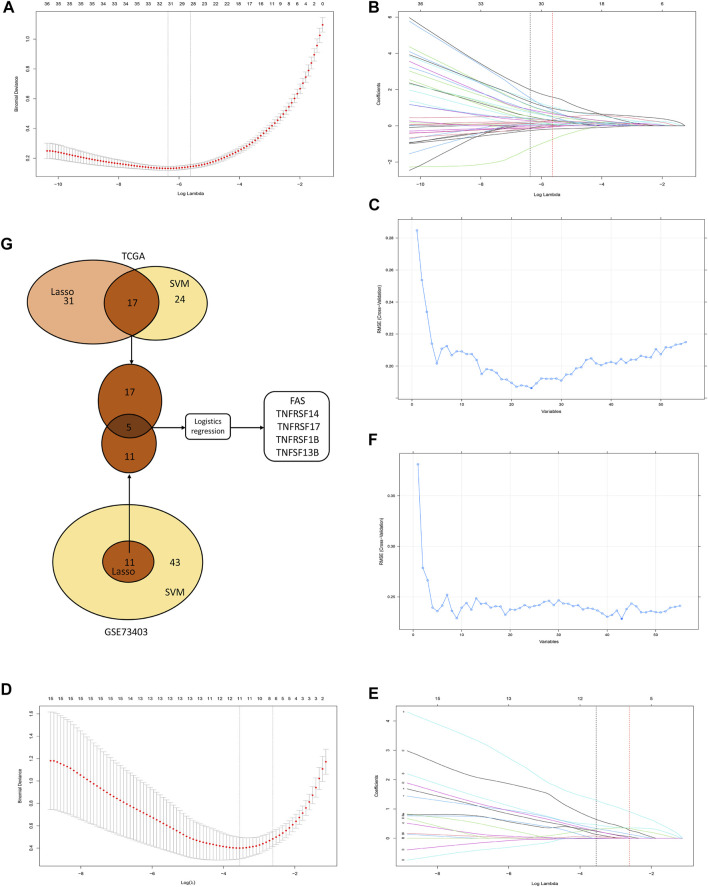
The selection of diagnostic markers from among candidate costimulatory molecule genes (CMGs). In Figures 7A, B, D, E, the lower abscissa is the log lambda value, while the upper abscissa is the number of CMGs with non-zero coefficient; the vertical axis represents the Least Absolute Shrinkage and Selection Operator (LASSO) coefficient of CMGs, and each curve shows the variation trajectory of the coefficients of each gene. **(A)** Determination of the number of CMGs with non-zero coefficients in TCGA dataset; **(B)** LASSO coefficient profiles of 31 candidate CMGs after the 10-fold cross-validation in TCGA dataset; **(C)** Support Vector Machine-Recursive Feature Elimination (SVM-RFE) method to identify markers in TCGA dataset; **(D)** Definition of the number of CMGs with non-zero coefficients in the GSE43403 dataset; **(E)** LASSO coefficient profiles of 11 candidate CMGs after the 10-fold cross-validation in the GSE73403 dataset; **(F)** SVM-RFE method to identify markers in the GSE73403 dataset; **(G)** Venn diagram presents the overlapping diagnostic markers identified by LASSO and SVM-REF algorithms.

The TCGA and GSE73403 were used to select the CMG biomarkers from 55 CMGs to identify the diagnostic biomarkers. In the TCGA dataset, we initially identified 31 biomarkers from 55 candidate CMGs using the LASSO logistic regression method (Figures 7A, B). The SVM-RFE machine learning algorithm was used to screen out 24 CMG biomarkers from all 55 candidates (Figure 7C). Then, we overlapped all diagnostic markers identified with the abovementioned algorithms in the TCGA, and 17 CMG markers remained (Figure 7G).

Then, in the GSE73403 dataset, 11 CMGs from 55 candidates were identified as putative diagnostic markers using the LASSO logistic regression method (Figures 7D, E). Meanwhile, the SVM-RFE machine learning algorithms were used to identify 43 CMGs (Figure 7F). Among them, 11 CMGs were overlapping (Figure 7G). Then, five candidate CMGs identified from the abovementioned two datasets were used in the logistic regression analysis to select the final diagnostic markers. Then, five CMGs (FAS, TNFRSF14, TNFRSF17, TNFRSF1B, and TNFSF13B) were considered the final diagnostic markers (Figure 7G). Supplementary Table S2 presents all CMGs analyzed in this phase. In addition, the heatmaps revealed that these five CMGs were up-regulated in “hot” tumor in TCGA, GSE73403 and GSE37745 datasets (Supplementary Figure S4).

### Development and validation of the diagnostic nomogram based on CMG biomarkers

**FIGURE 8:**
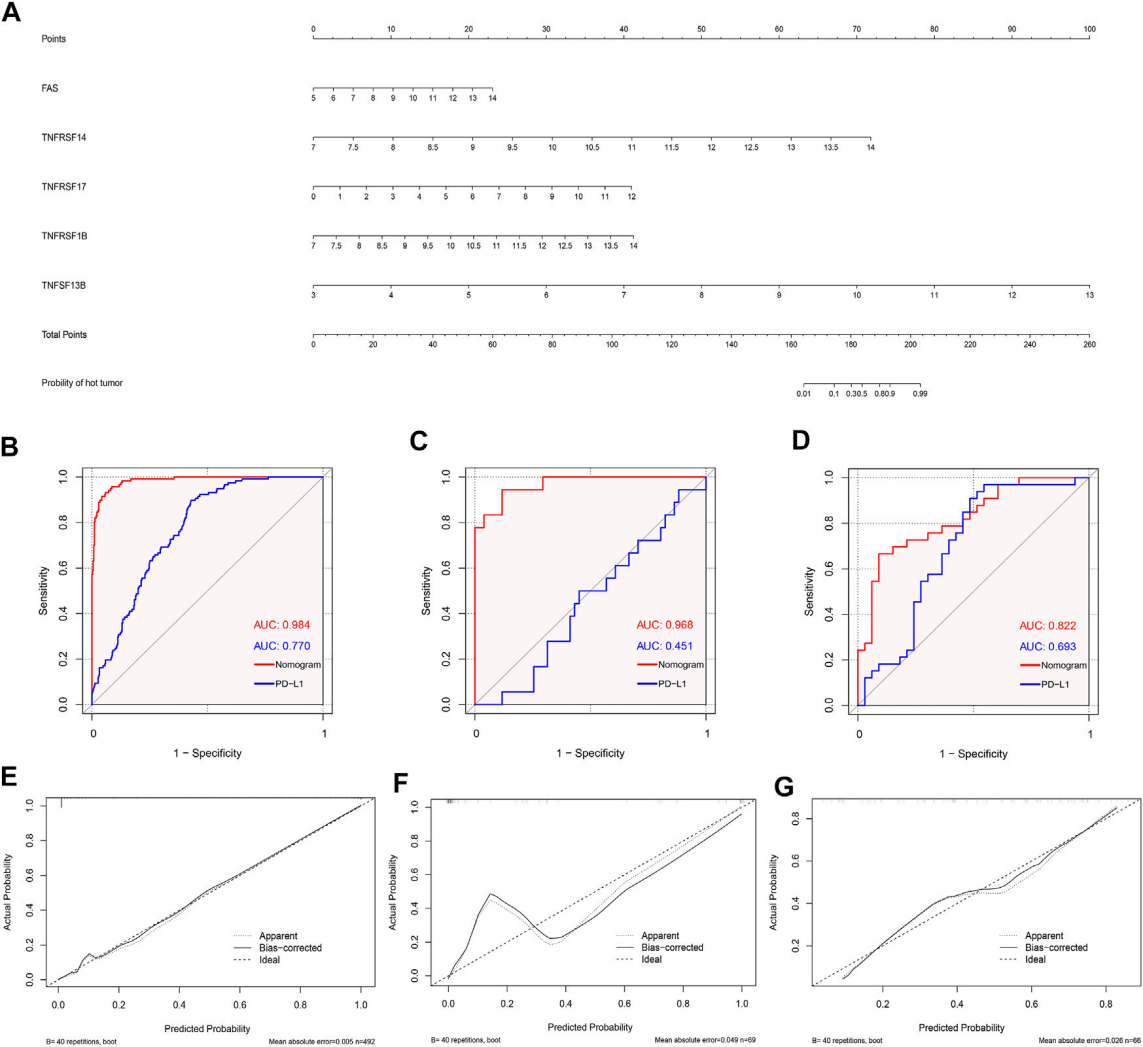
Development and validation of the diagnostic nomogram. **(A)** A nomogram for diagnosing individualized immune environment subclass. The receiver operating characteristic (ROC) curve for the diagnostic efficacy verification of nomogram and PD-L1 in TCGA **(B)**, GSE73403 **(C)**, and GSE37745 datasets **(D)**; The calibration plots of the diagnostic accuracy validation in three datasets **(E**–**G)**.

Based on the abovementioned CMG biomarkers, including FAS, TNFRSF14, TNFRSF17, TNFRSF1B, and TNFSF13B, we constructed a diagnostic nomogram for predicting individualized TIME subclass in patients with LUSC based on the TCGA training dataset (Figure 8A). According to our nomogram, each CMG marker could obtain a score in the point line, and the total score of each patient with LUSC could be calculated based on the biomarker expressions of the three CMGs. By identifying the total score using the hut tumor scale, the probability of patients with LUSC presenting with “hot” TIME could be predicted.

Furthermore, to evaluate the diagnostic performance and accuracy of the nomogram, we respectively drew the ROC and calculated the area under the curve (AUC) in the TCGA (Figure 8B), GSE73403 (Figure 8C), and GSE37745 (Figure 8D) datasets. The AUC of TCGA, GSE73403, and GSE37745 was 0.984, 0.968, and 0.882, respectively, which demonstrated a good diagnostic efficacy of the nomogram. Moreover, we compared the AUC between the nomogram and PD-L1 expression in the three datasets (Figures 8B–D). Results showed that the diagnostic performance of the nomogram was better than PD-L1 expression in three datasets (TCGA: 0.984 *versus* 0.770; GSE73403: 0.984 *versus* 0.451; GSE37745: 0.882 *versus* 0.693). Figures 8E–G show the calibration curves, whose axes represented the actual probability (*Y*-axis) and the probability predicted by the nomogram (*X*-axis). Results showed a favorable consistency between the virtual and predicted probability in all three datasets. Then, we investigated whether the combination of CMGs and PD-L1/TMB could enhance the predictive performance of the diagnostic model. We found the combination of CMGs and PD-L1 could enhance the predictive performance in three datasets but this improvement was not apparent (AUC, TCGA: 0.986; GSE73403: 0.980; GSE37745: 0.957). However, combination of CMGs, PD-L1 and TMB could not enhance the predictive performance in TCGA dataset (AUC, 0.986) (Supplementary Figure S5).

### Diagnostic value of the five CMGs in immunotherapy

**FIGURE 9:**
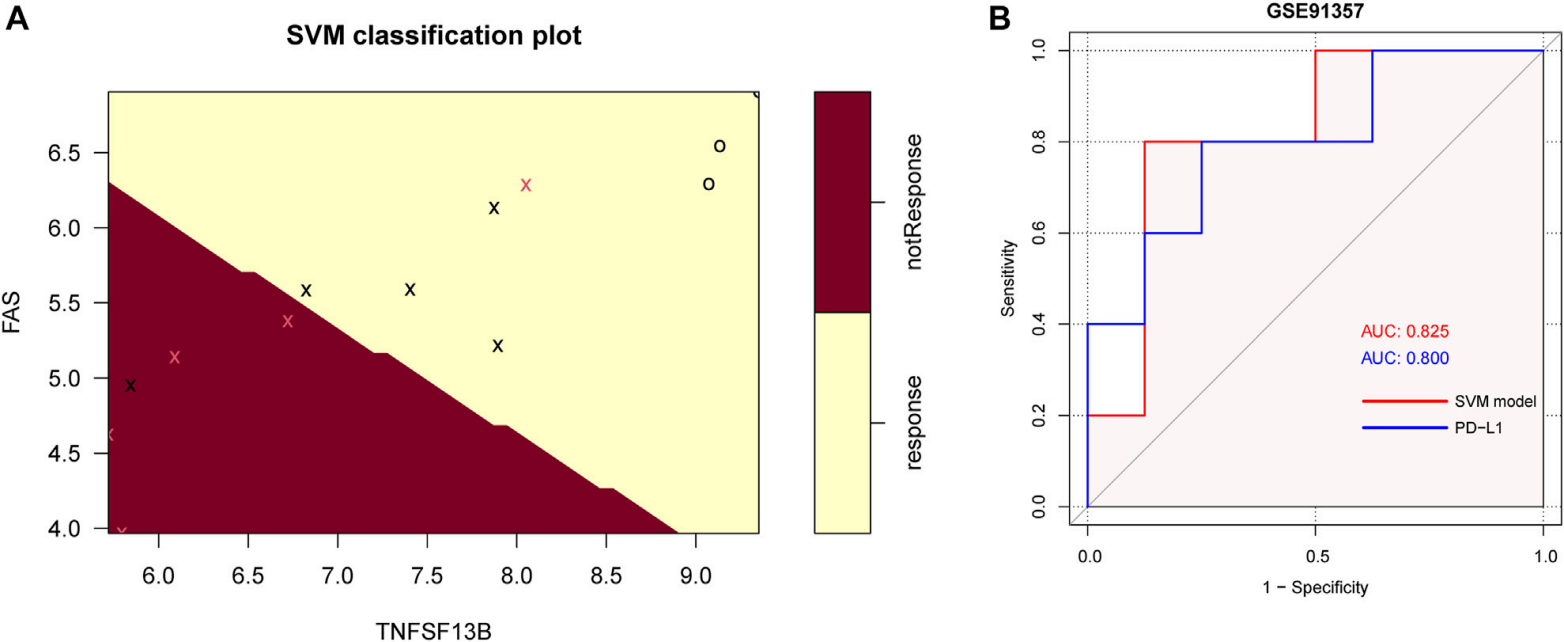
The SVM model of 5 CMGs for predicting response of immunotherapy in GSE93157. **(A)** The classification plot of the SVM model brown area: predicting probability of not response, yellow area: predicting probability of response, pink cross: actual not response, black cross or circle: actual response; **(B)** The receiver operating characteristic (ROC) curve for the diagnostic efficacy verification of SVM model and PD-L1 in GSE93157.

In the GSE93157 dataset, the SVM model based on the five CMGs demonstrated a high precision in predicting response to anti-PD1 therapy of LUSC patients. The response was correctly predicted in 11 of 13 (84.6%) patients (Figure 9A). The AUC of the SVM model was also higher than PD-L1 (0.825 *versus* 0.800) (Figure 9B).

## Discussion

In recent years, cancer immunotherapy has significantly improved. Moreover, owing to the development of ICIs, patients with LUSC without driver gene mutations presented with better survival outcomes and therapeutic landscape (Paz-Ares et al., 2018; Forde et al., 2021; Miller and Hanna, 2021; Zhao et al., 2021). However, only few patients with LUSC benefited from immunotherapy due to *de novo* or acquired resistance to ICIs (Insa et al., 2021; Shang et al., 2021). Increasing evidence showed that immune anergic is rendered by immunosuppression. Thus, T cells get exhausted, and immune cells cannot accurately recognize tumor antigen (Hanahan and Weinberg, 2011). In this study, we systematically investigated the clinical value of costimulatory molecules in predicting the TIME subclass among patients with LUSC. Initially, they were clustered based on their CMG expression pattern using the unsupervised clustering method. Next, patients in the three datasets were stratified into the “hot” and “cold” tumor subclasses according to differences in immune and stromal scores in the TIME among different clusters. Immune infiltration analyses revealed that “hot” tumors had a higher proportion of anti-tumor immune cells, such as CD8^+^ T cell, activated memory CD4^+^ T cell, gamma delta T cell, and M1 macrophage. The functional and pathway enrichment analysis further revealed that “hot” LUSC tumors were significantly enriched in the B and T cell receptor signaling pathways. Moreover, they were mainly associated with the regulation of T cell differentiation and activation and immune responses. Furthermore, we screened out five CMGs, including FAS, TNFRSF14, TNFRSF17, TNFRSF1B, and TNFSF13B. They were found to be diagnostic biomarkers based on an analysis using two machine learning algorithms (LASSO and SVM-RFE) and several bioinformatics. Subsequently, using the abovementioned CMG biomarkers, we established a diagnostic nomogram for predicting individualized TIME subclasses among patients with LUSC. In addition, whether our nomogram had good predictive efficacy and satisfactory clinical value was evaluated and validated in the TCGA and two independent validation datasets. Furthermore, the SVM model revealed the predictive value of these five CMGs in immunotherapy. Therefore, our CMG-based diagnostic nomogram could be a practical tool for stratifying the TIME subclasses of patients with LUSC and might provide useful therapeutic recommendations for ICI therapy. To the best of our knowledge, this is the first and the most comprehensive study that investigated the TIME subclass prediction value of CMGs among patients with LUSC.

Anti-PD-L1, anti-PD-1, or anti-CTLA4 antibodies can block the PD1-PD-L1 or the CD86/CTLA4 axes, which recover cytotoxic T-cell response against tumors (Fife and Bluestone, 2008). PD-L1 is the most widely used biomarker in immunotherapy. However, it still has some limitations (Doroshow et al., 2021; Mino-Kenudson et al., 2022). Recently, immune infiltration has been considered a novel biomarker for predicting the prognosis and response of patients receiving ICI therapy (Fridman et al., 2012; Gentles et al., 2015; Oliver et al., 2018; Zuo et al., 2020). Kirfel et al. compared the expression of PD-L1 and immune cell infiltration *via* immunohistochemical staining in 138 NSCLC samples (Kirfel et al., 2021). Results showed that PD-L1-positive tumors had “cold” immune infiltration in TIME. Meanwhile, PD-L1-negative tumors had “hot” immune infiltration in TIME. The poor predictive accuracy of PD-L1 can be explained by the separation of PD-L1 expression and immune infiltration status. In this study, our nomogram had a better performance in predicting “hot” tumors than PD-L1 alone.

Fas cell surface death receptor is encoded by the FAS gene, and it plays an important part in the apoptotic signal pathway in several cells. Fas ligand, which is the ligand of this receptor, is expressed restrictedly in immune cells (Zhang et al., 2005). The binding of the Fas ligand to the Fas receptor leads to cell apoptosis (Müschen et al., 2000). TNFRSF14 is transmembrane glycoprotein belonging to the TNF receptor family. The activation of TNFRSF14 participates in the process of immune cell survival and differentiation, promotes T cell proliferation and functions, and increases interferon production and the anti-tumor effect of NK cells (Marsters et al., 1997; Tamada et al., 2000; Šedý et al., 2013). TNFRSF17 is a B cell maturation antigen, which also belongs to the TNF receptor family. It plays an essential role in B cell proliferation and differentiation into plasma cells and prolonging the survival of plasma cells (Xu and Lam, 2001; Benson et al., 2008). TNFRSF1B, a TNF receptor, is associated with T-cell responses, and it participates in protective immunity, inflammatory disease, and autoimmune diseases (So and Ishii, 2019). TNFSF13B, also known as B-cell-activating factor, is a member of the TNF family, and it is expressed by dendritic cells, monocytes, and macrophages (Batten et al., 2000). Moreover, it is necessary in B cell survival and maturation, and it stimulates T cells by costimulating the signal pathway (Huard et al., 2001).

Previous studies have presented immune-related biomarkers for predicting the prognosis of patients with lung cancer, which is closely correlated with TIME. Zhang et al. established a five CMG signature based on 10 datasets, and patients were then divided into the high- and low-risk groups. Results showed that TIMEs differed in the low-risk groups (Zhang et al., 2020). Peng et al. used multiplex immunofluorescence to investigate 26 types of immune cells and clustered three groups of patients with discrepant TIME. Further, they identified a biomarker that included six types of cells for predicting survival (Peng et al., 2021). However, the abovementioned studies used the prognostic model instead of the diagnostic model. To date, there is still no diagnostic model for predicting “cold” and “hot” tumors among patients with LUSC. Therefore, we developed this diagnostic tool for predicting the individual status of TIME, which is a potential biomarker for ICI therapy, thereby providing immunotherapeutic guidance.

Based on the abovementioned five CMGs, we established a diagnostic nomogram based on the TCGA set for patients with LUSC. Results revealed that high FAS, TNFRSF14, TNFRSF17, TNFRSF1B, and TNFSF13B expressions are associated with a high probability of “hot” tumor. Furthermore, the predictive accuracy of the diagnostic nomogram was high in the TCGA (ROC = 0.984) GSE73403 (ROC = 0.968), and GSE37745 (ROC = 0.822) datasets.

Despite the abovementioned advantages, the current study had several limitations. First, all datasets were obtained from public databases, and the power and credibility of our findings could be strengthened by further performing validations in a real-world cohort. Second, the underlying mechanism of these three CMGs biomarkers was not explored, and a deep understanding of their mechanism of action is important for identifying novel therapeutic targets in the future. Third, the diagnostic accuracy and clinical value of CMGs among patients with LUSC was investigated using machine learning algorithms and bioinformatic methods alone. Therefore, a bias might be inevitable. Therefore, an experimental confirmation using corresponding small-molecule inhibitors or multicenter clinical studies must be performed. Fourth, a diagnostic nomogram was developed using TCGA data including samples from the United States, its diagnostic performance was validated using two other independent GEO datasets comprising cases from China and Sweden. Therefore, the use of our model in patients with other background characteristics or those from different regions should be taken with caution due to the heterogeneity of patients with LUSC.

In conclusion, using machine learning algorithm and bioinformatics, we comprehensively parsed the expression patterns of CMGs and recognized two distinct TIME subclasses among patients with LUSC. Five CMGs, including FAS, TNFRSF14, TNFRSF17, TNFRSF1B, and TNFSF13B, were found to be diagnostic markers. Then, a novel diagnostic nomogram for predicting individual TIME status was developed based on these CMGs. This nomogram had a good predictive accuracy. Thus, it could be used to identify patients who may benefit more from ICI therapy.

## Data Availability

Publicly available datasets were analyzed in this study. This data can be found here: TCGA databases (https://tcga-data.nci.nih.gov/tcga/) and the GEO databases (https://www.ncbi.nlm.nih.gov/geo/).

## Supplementary material

SUPPLEMENTARY FIGURE S1 Comparison of tumor purity among different LUSC patient clusters. The comparison of tumor purity in TCGA **(A)**; GSE73403 **(B)**; and GSE37745 datasets **(C)**.

SUPPLEMENTARY FIGURE S2 Comparison of tumor purity between different immune environment subclasses. Significant differences in tumor purity between the “cold” and the “hot” tumor groups in TCGA **(A)**; GSE73403 **(B)**; and GSE37745 datasets **(C)**.

SUPPLEMENTARY FIGURE S3 The violin plot based on MCP-counter depicts infiltration disparities among immune cell types between the “hot” tumor group (red) and the “cold” tumor group (blue) in TCGA **(A)**, GSE73403 **(B)**, and GSE37745 datasets **(C)**.

SUPPLEMENTARY FIGURE S4 The heatmap showed the expression of the five CMGs between “hot” and “cold” tumor in TCGA **(A)**, GSE73403 **(B)**, and GSE37745 datasets **(C)**.

SUPPLEMENTARY FIGURE S5 The receiver operating characteristic (ROC) curve for the diagnostic efficacy verification of nomogram plus PD-L1 in TCGA **(A)**, GSE73403 **(B)**, and GSE37745 datasets **(C)**. The ROC curve for the diagnostic efficacy verification of nomogram plus PD-L1 and TMB in TCGA dataset **(D)**.
