# Supplementary figures and images for: A novel diagnostic model for predicting immune microenvironment subclass based on costimulatory molecules in lung squamous carcinoma
Source: Front Genet. 2022 Dec 14;13:1078790. doi: 10.3389/fgene.2022.1078790 (PMC9795004; doi:10.3389/fgene.2022.1078790)

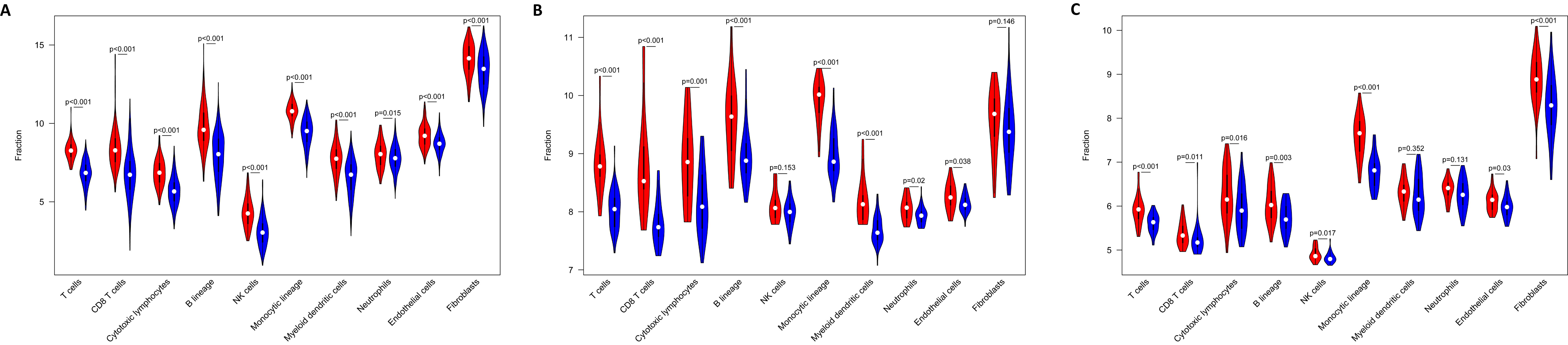

Supplement: Supplementary file 3 [file Image3.TIF]

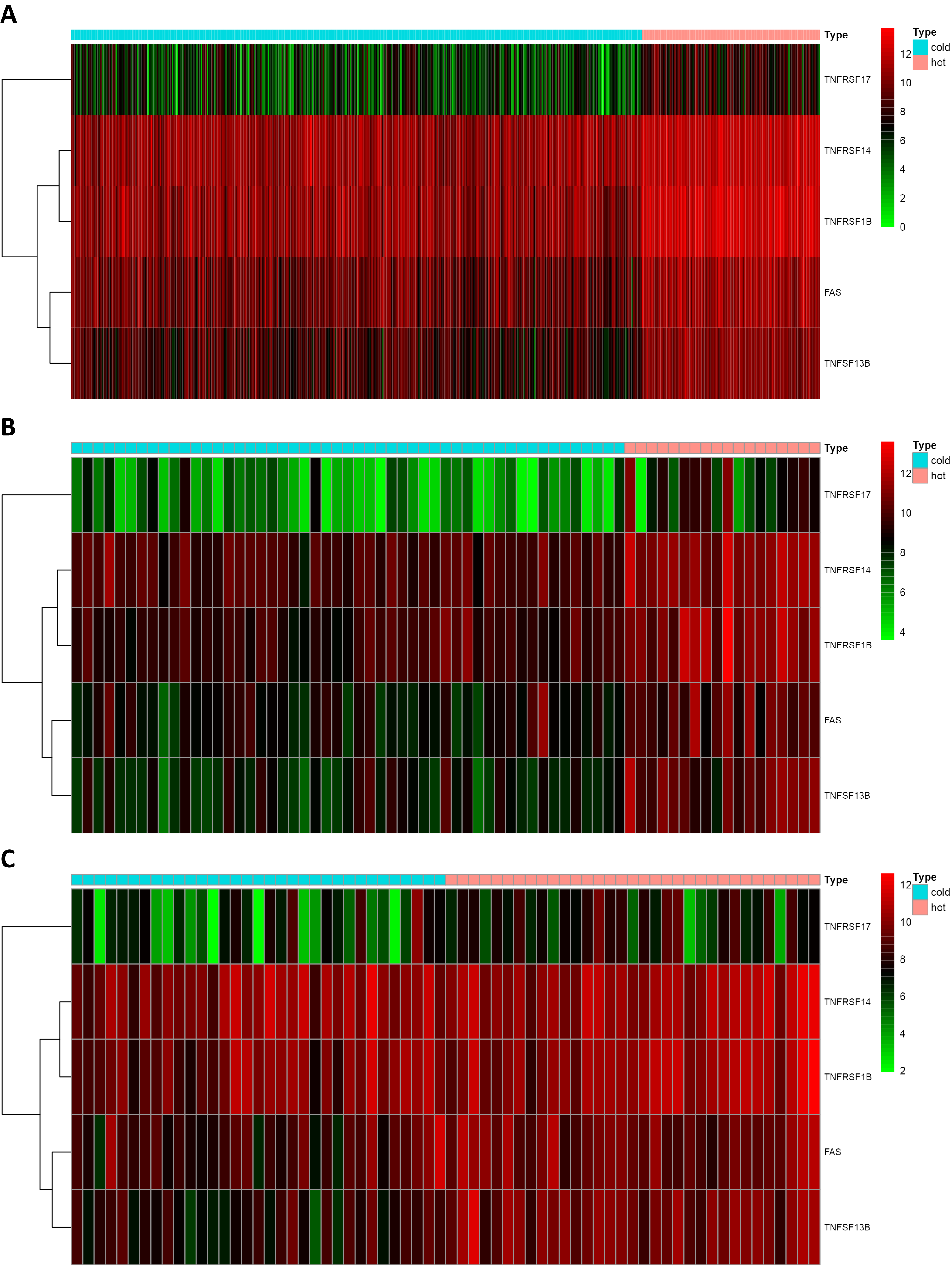

Supplement: Supplementary file 4 [file Image4.TIF]

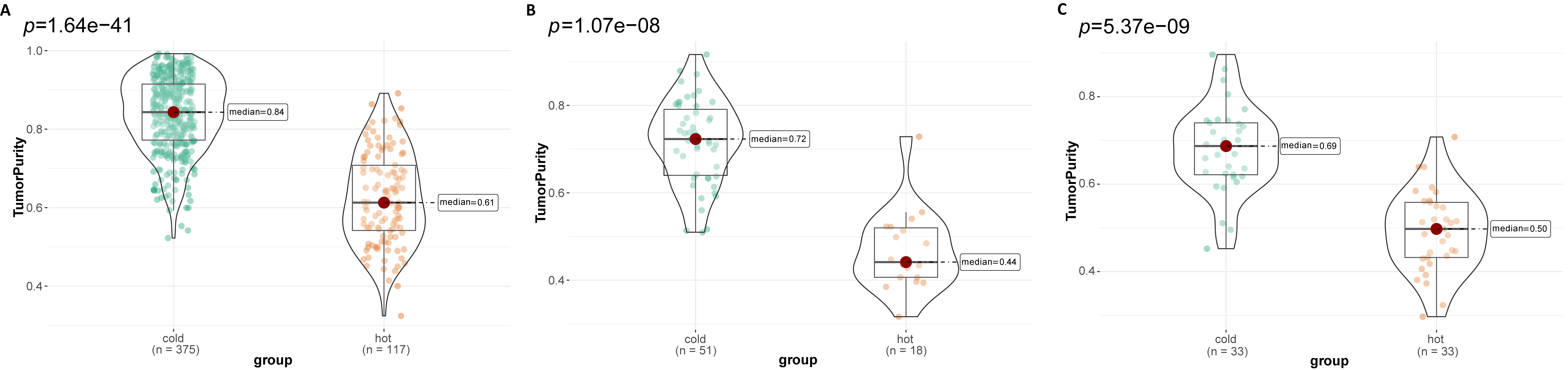

Supplement: Supplementary file 5 [file Image2.TIF]

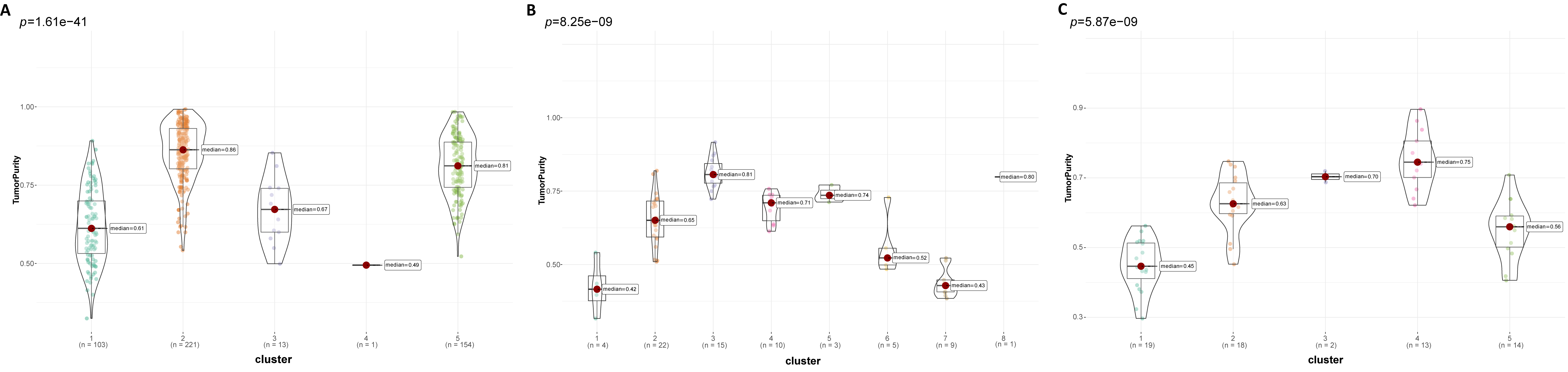

Supplement: Supplementary file 6 [file Image1.TIF]

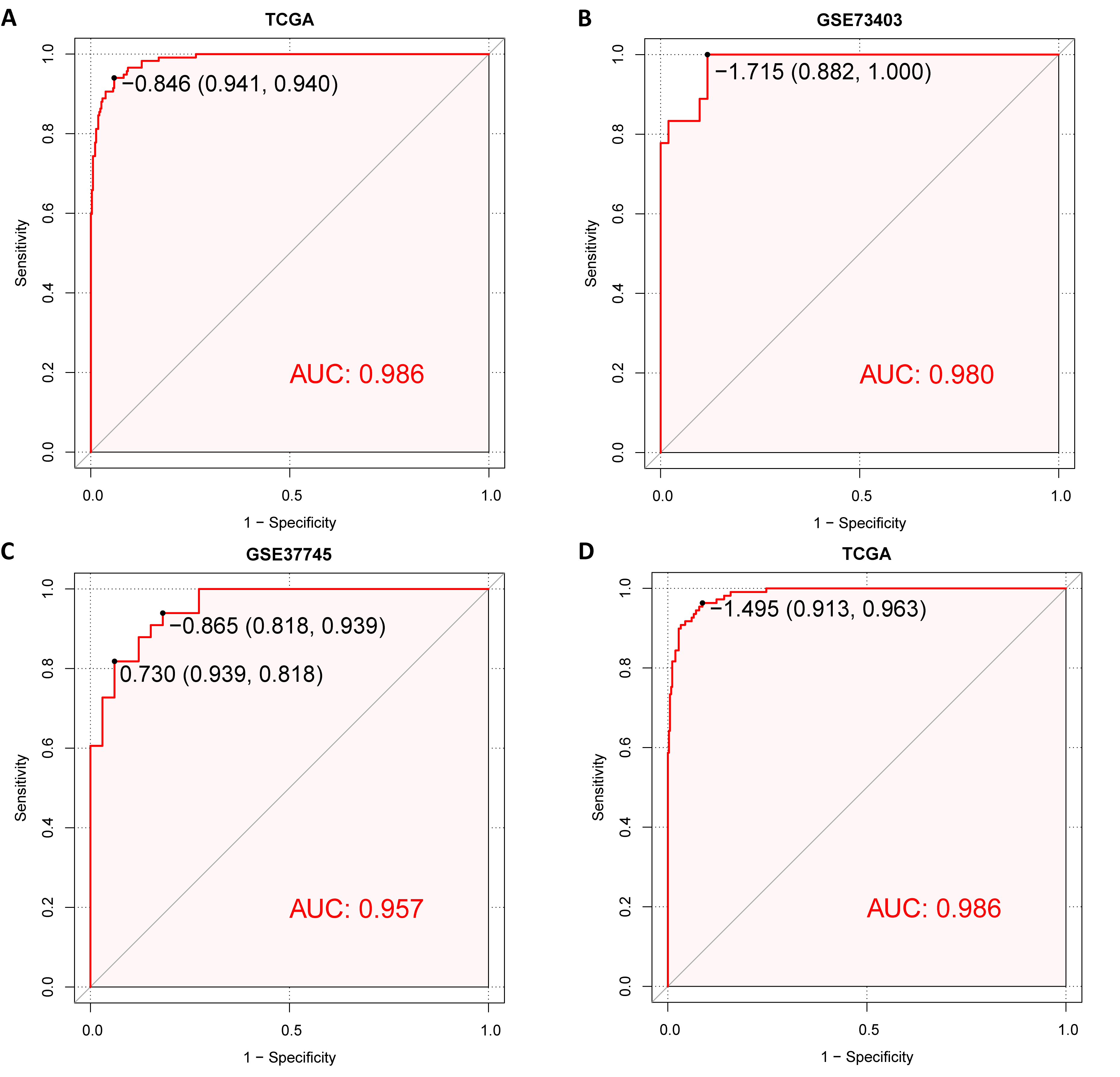

Supplement: Supplementary file 7 [file Image5.TIF]
